# Urban-Rural Disparities in Sensory Disability and Fatal Car Crashes Among Older Adults in Mississippi

**DOI:** 10.1093/geroni/igaf122.3414

**Published:** 2025-12-31

**Authors:** Wuyi Dong, Yan-Jhu Su, Taylor Jansen, Elizabeth Dugan

**Affiliations:** University of Massachusetts Boston, Boston, Massachusetts, United States; The Pennsylvania State University, University Park, Massachusetts, United States; University of Massachusetts Boston, Boston, Massachusetts, United States; University of Massachusetts Boston, Boston, Massachusetts, United States

## Abstract

Understanding urban-rural disparities in sensory disabilities and traffic safety is essential for developing targeted interventions that enhance mobility and quality of life among older adults. Using data from the 2023 Mississippi Healthy Aging Data Reports (HADR), this cross-sectional study examines county-level cases of fatal car crashes involving individuals aged 60 and older and the rates of self-reported sensory difficulties (vision and hearing impairments) in Mississippi. Analyzing 82 counties (17 urban, 65 rural), we identify variations in fatal crash (urban: 15.76; rural: 7.88; state average: 9.51) and sensory difficulties (state averages—hearing: 15.5%, vision: 8.7%; urban ranges—hearing: 8.5%–27.7%, vision: 3.1%–15.1%; rural ranges—hearing: 5.4%–35.0%, vision: 0%–20.6%). Bivariate mapping overlays these data to pinpoint counties with both high fatal crash rates and sensory impairment prevalence. This exploratory analysis offers critical insights into the intersection of sensory disabilities and traffic safety, guiding policymakers and service providers in prioritizing transportation interventions and support services for older adults in urban and rural communities.

